# Integrin α10, a Novel Therapeutic Target in Glioblastoma, Regulates Cell Migration, Proliferation, and Survival

**DOI:** 10.3390/cancers11040587

**Published:** 2019-04-25

**Authors:** Matilda Munksgaard Thorén, Katarzyna Chmielarska Masoumi, Cecilia Krona, Xiaoli Huang, Soumi Kundu, Linnéa Schmidt, Karin Forsberg-Nilsson, Marcus Floyd Keep, Elisabet Englund, Sven Nelander, Bo Holmqvist, Evy Lundgren-Åkerlund

**Affiliations:** 1 Xintela AB, Medicon Village, SE-223 81 Lund, Sweden; matilda@xintela.se (M.M.T.); katarzyna@xintela.se (K.C.M.); xiaoli@xintela.se (X.H.); linnea@clin.au.dk (L.S.); 2 Department of Immunology, Genetics and Pathology, Science for Life Laboratory, Rudbeck Laboratory, Uppsala University, SE-751 85 Uppsala, Sweden; cecilia.krona@igp.uu.se (C.K.); soumi.kundu@igp.uu.se (S.K.); karin.nilsson@igp.uu.se (K.F.-N.); sven.nelander@igp.uu.se (S.N.); 3 Department of Neurosurgery, Sanford Brain and Spine Institute, Fargo, ND 58103, USA; Department of Surgery, School of Medicine, University of North Dakota, Fargo, ND 58102, USA; mkeep@maasbiolab.com; 4 Neuropathology Lab, Division of Oncology and Pathology, Department of Clinical Sciences, Lund University, SE-221 84 Lund, Sweden; elisabet.englund@med.lu.se; 5 ImaGene-iT AB, Medicon Village, SE-223 81 Lund, Sweden; bo@imagene-it.se

**Keywords:** glioblastoma (GBM), glioma, integrin α10, antibody–drug conjugate (ADC), saporin

## Abstract

New, effective treatment strategies for glioblastomas (GBMs), the most malignant and invasive brain tumors in adults, are highly needed. In this study, we investigated the potential of integrin α10β1 as a therapeutic target in GBMs. Expression levels and the role of integrin α10β1 were studied in patient-derived GBM tissues and cell lines. The effect of an antibody–drug conjugate (ADC), an integrin α10 antibody conjugated to saporin, on GBM cells and in a xenograft mouse model was studied. We found that integrin α10β1 was strongly expressed in both GBM tissues and cells, whereas morphologically unaffected brain tissues showed only minor expression. Partial or no overlap was seen with integrins α3, α6, and α7, known to be expressed in GBM. Further analysis of a subpopulation of GBM cells selected for high integrin α10 expression demonstrated increased proliferation and sphere formation. Additionally, siRNA-mediated knockdown of integrin α10 in GBM cells led to decreased migration and increased cell death. Furthermore, the ADC reduced viability and sphere formation of GBM cells and induced cell death both *in vitro* and *in vivo*. Our results demonstrate that integrin α10β1 has a functional role in GBM cells and is a novel, potential therapeutic target for the treatment of GBM.

## 1. Introduction

Glioblastomas (GBMs) belong to the group of high-grade gliomas that are among the most invasive and malignant brain tumors in adults, representing about 15% of all diagnosed brain tumors and affecting three individuals per 100,000 per year [1]. Primary brain tumors are classified into several different types and subtypes and since the publication of the World Health Organization classification of central nervous system tumors in 2016, both histological and molecular parameters are used to define a tumor entity [2]. GBMs have poor prognoses and may be markedly resistant to both radiotherapy and chemotherapy, and no curative treatment is yet available. Standard treatment consists of combinations of maximal neurosurgical resection, reducing tumor burden and pressure on the brain, radiation therapy, and concurrent and continual chemotherapy with temozolomide. Without treatment, the average survival time is just four months from diagnosis. Even with treatment, relapse is anticipated, and with maximal, existing treatment, patient survival is still only about 15 months [3,4,5]. Thus, there is a significant need to improve and find new treatment strategies targeting this form of aggressive tumor.

Genetic and phenotypic heterogeneity is a common feature of GBM, which constitutes a major challenge for the development of an effective therapy [6,7,8]. In recent years, increasing evidence suggests that the major culprit for cancer recurrence and treatment resistance is cancer stem-like cells. Similar to normal stem cells, cancer stem-like cells have self-renewal, anchorage-independent growth, and pluripotent differentiation abilities [9]. Cancer stem-like cells have been implicated in several human cancers, including gliomas [10,11,12,13,14]. The high plasticity of GBM cells has been suggested to be another important driver of tumor heterogeneity and dedifferentiation of tumor cells into a stem-like state [15]. The glioma stem-like cells are generally believed to express stemness and self-renewal markers, such as Sox2 and Nestin [16,17,18]. Sox2 and Nestin are enriched in glioma stem-like cells and maintain the cells’ stemness property and continued tumorigenicity. Expression of Sox2 identifies patients with the worst clinical outcomes [19]. With growing evidence that GBM arises, progresses, and recurs from stem-like cells, novel therapies targeting this subpopulation of cancer cells provide new possibilities for extending patient survival.

Rapid invasion of the surrounding normal brain tissue is a main characteristic of malignant and recurrent GBM tumors. The grade of invasiveness is closely related to the prognosis [3]. Interactions between extracellular matrix (ECM) and integrin receptors on tumor cells have been shown to play a key role in tumor cell processes, such as cell migration and proliferation, as well as in angiogenesis [20,21]. Integrins are heterodimeric cell surface receptors, consisting of an α and β chain that link the ECM to the cytoskeleton and connect mechanical cues with intracellular signaling cascades. Integrin α10β1 was discovered by our group and originally identified as a type II collagen-binding receptor on chondrocytes [22]. The expression of α10β1 in normal tissues is restricted and mainly confined to cartilage-containing tissues [23,24]. We have reported that mice lacking the integrin α10β1 have defects in the cartilaginous growth plate and consequently develop growth retardation of the long bones [25]. We have also reported that integrin α10β1 is present on mesenchymal stem cells (MSCs) and that the expression correlates with the chondrogenic differentiation potential of MSCs [26]. Recently, we demonstrated that antibodies to integrin α10 identify and select a subfraction of cells from MSC preparations with improved trilineage differentiation potential [27]. Here, we report that integrin α10β1 is highly expressed in GBM cells, both in cultured GBM cell lines with stem cell characteristics and in patient GBM tumor tissue samples.

Little is known about integrin α10β1 in cancer, but recent studies suggest that integrin α10β1 may play an important role in the development and progression of certain tumors. For example, it has been shown that integrin α10 expression is up-regulated in malignant melanoma cells compared with primary melanocytes [28]. This study also demonstrated that melanoma cells treated with either an inhibitory antibody to integrin α10 or an antisense construct downregulating α10 expression had reduced migratory potential, thus suggesting a role for integrin α10 in melanoma cell migration. Furthermore, integrin α10β1 has been shown to be an important receptor on α-smooth muscle actin (α-SMA)-expressing stromal cells in human ovarian tumors by binding to the HU177 cryptic collagen epitope [29]. Additionally, the integrin α10 gene (*ITGA10*) has been shown to be the most significant gene associated with disease-specific death and distant metastasis in myxofibrosarcoma [30]. In the present study, we demonstrate that integrin α10β1 is highly expressed in high-grade glioma tumor tissue and patient-derived GBM cell lines and that it plays a role in the viability and migration of GBM cells. Furthermore, in a xenografted mouse model, we demonstrate that treatment with an antibody–drug conjugate (ADC), composed of the integrin α10 antibody conjugated to the potent cytotoxin saporin [31], anti-α10-SAP, induces cell death of these tumor cells. Our data indicate that ADC therapy targeting α10β1 may represent a new strategy to treat glioblastoma and other high-grade gliomas.

## 2. Results

Integrin α10β1 is expressed on cells in human glioma tissues and in GBM cell lines.

### 2.1. Expression of Integrin α10β1 Is Increased in High-Grade Gliomas

**Figure 1:**
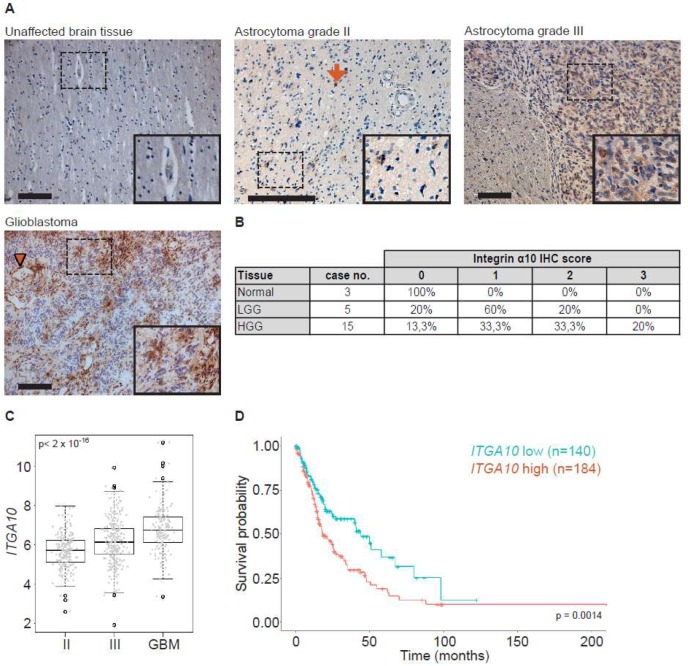
Integrin α10 is expressed in human glioma tissue. (**A**) Immunohistochemical evaluation of integrin α10 expression (brown) in morphologically benign brain tissue and in tumors defined as human astrocytoma grades II and III and glioblastoma (GBM) specimens using a polyclonal antibody directed against the cytoplasmic tail of integrin α10. The arrow indicates integrin α10-positive cells in astrocytoma grade II tissue, and the arrowhead in GBM tissue indicates α10 expression surrounding a blood vessel. Scale bars represent 100 µm in unaffected brain tissue and in grade III tissue, 200 µm in grade II tissue, and 50 µm in GBM tissue. Boxed areas are shown at a higher magnification in the lower right images. Immunohistochemical scoring of labeling intensities for integrin α10-immunoreactive cells in normal and glioma [low-grade (LGG) and high-grade (HGG)] tissue is summarized in panel (**B**). Scoring of the labeling intensity of the tissues was 0 (negative), 1 (weak), 2 (moderate), and 3 (strong). (**C**) Normalized TCGA (The Cancer Genome Atlas) gene expression data (IlluminaHiSeq, San Diego, CA, USA) for low-grade gliomas and GBM, and clinical information on the grade of low-grade gliomas was used to stratify patients into astrocytoma grades II and III, and GBM. All statistical analyses were performed using one-way ANOVA and the non-parametric alternative (Kruskal–Wallis test). A statistically significant difference was found between the three groups (*p*-value ˂ 2 × 10^−16^). (**D**) Kaplan–Meier curve comparing the overall survival probability of glioma patients (astrocytoma grade II, *n* = 59; astrocytoma grade III, *n* = 116; and GBM *n* = 149) with low vs. high *ITGA10* mRNA levels. Patients were divided into high and low *ITGA10* expression groups based on the median cut off for the survival analysis by the Kaplan–Meier method and log-rank test. *p*-value = 0.0014.

Immunolabeling of human glioma tissue samples with antibodies directed against integrin subunit α10 demonstrated a broad distribution and distinct cellular expression of integrin α10β1 in high-grade gliomas (astrocytoma grade III and GBM) (Figure 1A). The integrin expression was found on dispersed single cells, on small cell clusters in the vicinity of blood vessels, and on large cell clusters within the tumor mass (Figure 1A). In contrast, only a few cells dispersed in the tissue expressed integrin α10β1 in low-grade gliomas (astrocytoma grade II), as indicated by the arrow (Figure 1A). Control tissue, i.e., brain tissue with normal brain morphology, contained no or very few, weakly labeled α10β1-expressing cells (Figure 1A). Correspondingly, by scoring the intensity of the integrin α10β1 immunoreactivity (from 0 to 3) in glioma tissues at different stages, we found an increased intensity of integrin α10β1 expression in the high-grade gliomas compared with the low-grade gliomas and normal tissue (Figure 1B).

When we analyzed the gene expression of *ITGA10* using the cancer genome atlas (TCGA) dataset for low- (II) and high-grade gliomas (III and GBM), we found that *ITGA10* expression significantly increased with the increasing tumor grade (Figure 1C), supporting the protein expression results from the glioma tissues. Additionally, the TCGA datasets for deceased glioma patients (including astrocytoma grades II, III, and GBM) revealed a significantly better overall survival probability when tumors expressed lower levels of integrin α10 (Figure 1D). 

### 2.2. Patient-Derived GBM Cell Lines Express Integrin α10β1 

**Figure 2:**
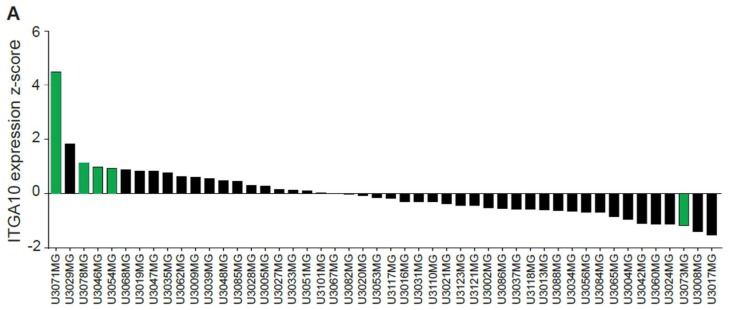

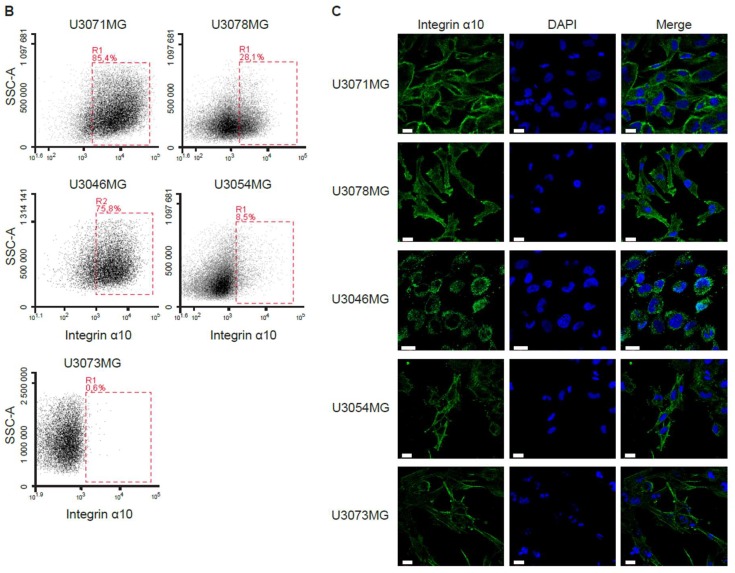
Integrin α10 is expressed in patient-derived GBM cell lines. (**A**) *ITGA10* gene expression from the Human Glioblastoma Cell Culture (HGCC) database, including 48 cell lines plotted as z-scores. Cell lines marked with green color were selected for further characterization. (**B**,**C**) Detection of integrin α10 protein expression on patient-derived GBM cell lines U3071MG, U3078MG, U3046MG, U3054MG, and U3073MG. (**B**) Flow cytometry profiles of integrin α10 and the fluorescence intensity of cells labeled with the antibody directed against α10 plotted against side scatter. (**C**) Immunofluorescence analysis of labeled integrin α10 protein, 4′,6-diamidino-2-phenylindole (DAPI) staining of cell nuclei and a merged image (Merge). Scale bars represent 20 µm.

Gene expression analysis of 48 well-characterized GBM cell lines from patient surgical samples (Human Glioblastoma Cell Culture (HGCC)) revealed expression of *ITGA10* in all tested cell lines, although the expression levels varied between the different cell lines (Figure 2A). We further examined five cell lines (U3071MG, U3078MG, U3046MG, U3054MG, and U3073MG) with high or low *ITGA10* expression using flow cytometry (Figure 2B) and immunofluorescence analysis (Figure 2C). Cell line U3071MG, with the highest gene expression level of *ITGA10*, also expressed the integrin α10 protein to a high degree, whereas the other cell lines showed lower expression levels as determined by flow cytometry (Figure 2B). Immunofluorescence staining demonstrated typical, cell-surface expression of the integrin α10 protein on all cells, albeit with different intensity. Cell line U3071MG appeared to have the highest expression level of integrin α10 protein (Figure 2B,C). 

### 2.3. Phenotypic Characterization of the Integrin α10β1-Expressing GBM Cells 

**Figure 3:**
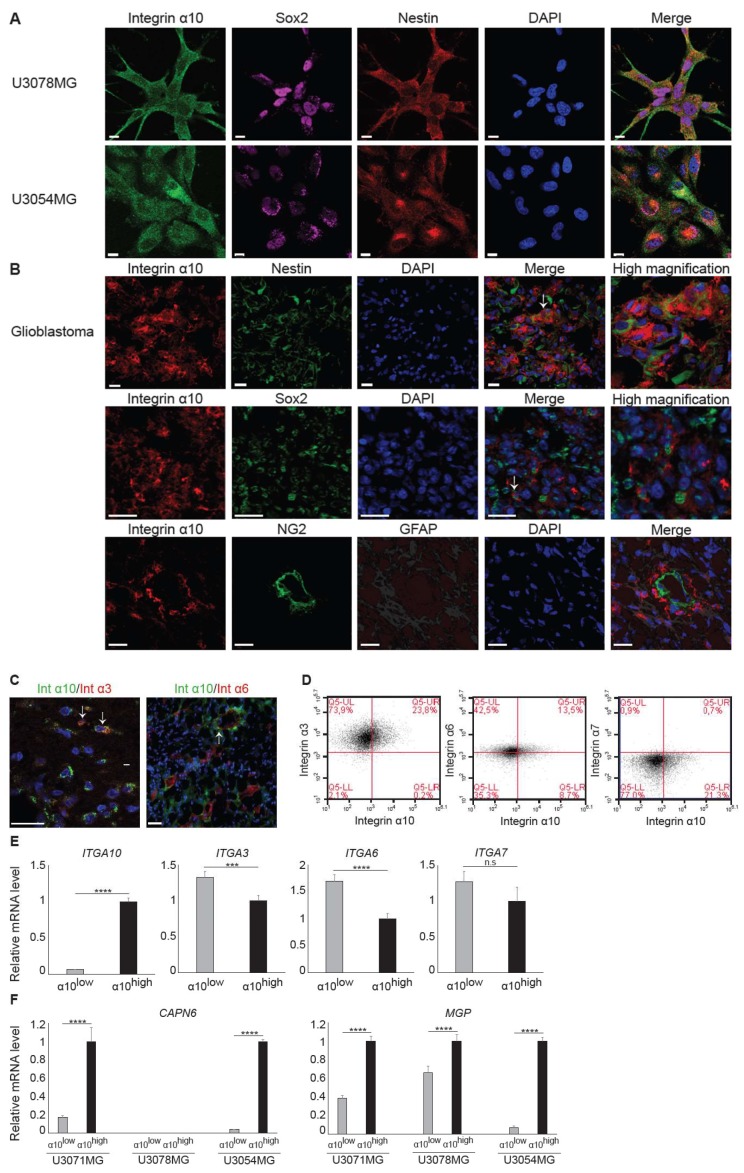
Integrin α10^high^-expressing cells define a subpopulation of GBM cells. (**A**) Representative images of U3078MG and U3054MG GBM cells, triple immunofluorescence labeled for integrin α10, Sox2, Nestin, DAPI staining of cell nuclei, and a merged image (Merge). Scale bars represent 10 µm. (**B**) Representative images of immunofluorescence labeling for integrin α10, Nestin, Sox2, NG2, GFAP, DAPI staining of cell nuclei in human GBM tissue, and a merged image (Merge). White arrows point to the expression of the markers in the same cells. Scale bars represent 20 µm. (**C**) Merged images of integrin α10 (Int α10) and integrin α3 (Int α3) or integrin α6 (Int α6) and DAPI staining of cell nuclei in human GBM tissue. White arrows point to the co-expression of the markers. Scale bars represent 20 µm. (**D**) Dot plots showing flow cytometry data for co-expression of integrin α10 and integrin α3, α6 or α7 on U3054MG cells. (**E**) Expression of *ITGA10, ITGA3, ITGA6*, and *ITGA7* mRNA in α10^high^- and α10^low^-sorted U3054MG cells, as measured by qRT-PCR. *n* ≥ 3 from two independent experiments. The mRNA expression is normalized against α10^high^. (**F**) The two genes *CAPN6* and *MGP*, shown to be up-regulated in U3071MG, U3078MG, and U3054MG cells in the microarray data, were selected for analysis by qRT-PCR. *n* ≥ 5 from two independent experiments. The mRNA expression is normalized against α10^high^ for each cell line. Error bars show the SD and the unpaired two-tailed Student´s t-test was performed, where *** *p* ˂ 0.00,1 and **** *p* ˂ 0.0001. n.s.: not significant.

The HGCC glioblastoma cell lines were cultured under neural stem cell conditions, leading to the enrichment of a stem-like cell population that expresses commonly used stem cell lineage markers [32]. Immunofluorescence analysis of cell lines U3078MG and U3054MG demonstrated cellular co-expression between integrin α10β1 and the intracellular markers Sox2 and Nestin (Figure 3A). Patient tumor tissue samples demonstrated a partial, cellular colocalization of integrin α10β1 and Nestin (1‒10% of the integrin α10β1 cells), while fewer of the integrin α10-expressing cells also expressed Sox2 (Figure 3B). Additionally, we found colocalization of integrin α10β1 and the astrocyte marker GFAP (Glial fibrillary acidic protein) (10‒30%) on the tumor cells but no colocalization with the pericyte marker NG2 (Neural/Glial Antigen 2). 

To further explore the phenotype of the integrin α10β1-expressing GBM cells, we investigated co-expression with integrin subunits α3, α6, and α7, all of which have been described as being expressed by stem cell-like GBM cells [33,34,35]. We found a partial, cellular colocalization of integrin α10 and α3 or α6 in human GBM tissues (Figure 3C), while integrin α7 was not detected in analyzed tissues. As judged by flow cytometry, almost all of the integrin α10-expressing U3054MG cells expressed integrin α3, whereas only a partial overlap between integrin α10 and integrin α6 was found. Very few or none of the integrin α10 GBM cells expressed integrin α7 (Figure 3D). To further investigate the correlation between integrin α10β1 and the other analyzed integrins, U3054MG cells were sorted into high α10 intensity (α10^high^) and low α10 intensity (α10^low^) populations using fluorescence-activated cell sorting (FACS). We found that mRNA expression of *ITGA3* and *ITGA6* were all higher in the population of U3054MG cells with low intensity of integrin α10 (Figure 3E). There was no significant difference in the expression of *ITGA7;* however, the mRNA levels were very low (C_t_ levels above 33). These results suggest that integrin α10^high^-expressing cells may define a specific population of GBM cells. 

For a better understanding of the phenotype of this subpopulation of GBM cells, gene expression analysis was performed on U3071MG, U3078MG, and U3054MG cells sorted into integrin α10^high^ and α10^low^ populations. Notably, amongst the top 20 genes up-regulated in the α10^high^-expressing cells, eight are involved in cell migration and/or invasion (Table S1). Validation of the microarray data was performed by measuring the mRNA levels of two of the most up-regulated genes, *CAPN6* (calpain 6*)* and *MGP* (matrix Gla protein) using qRT-PCR (Figure 3F). The expression of *CAPN6* was very low in U3078MG cells, which may reflect the heterogeneity of the cell lines. These data support that integrin α10^high^-expressing GBM cells represent a population of the GBM cells involved in migration and invasion.

### 2.4. Functional Characterization of the Integrin α10β1-Expressing GBM Cells

#### 2.4.1. Integrin α10β1 Mediates Adhesion to Collagen and Laminin

**Figure 4:**
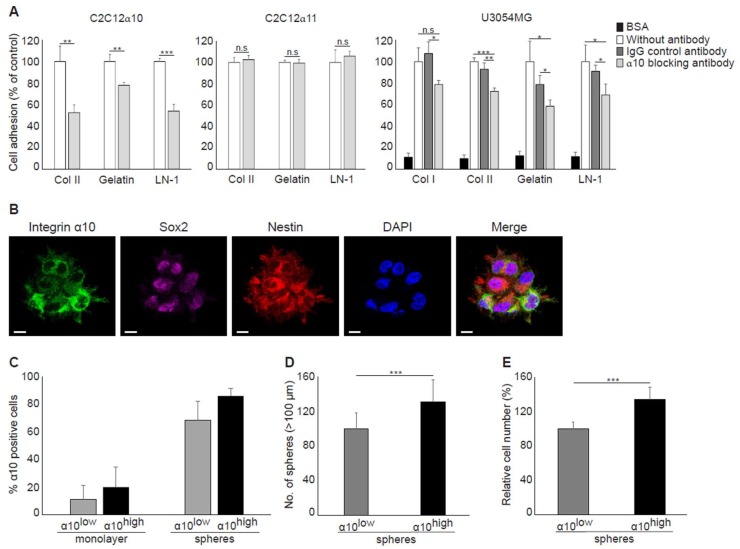
Integrin α10β1 mediates adhesion to collagen and laminin and promotes sphere formation and proliferation. (**A**) Adhesion of mouse myoblast cells C2C12 transduced with integrin α10 vector (C2C12α10) or integrin α11 vector (C2C12α11) and GBM U3054MG cells to dishes pre-coated with collagen I (Col I), collagen II (Col II), gelatin, or laminin-111 (LN-1) for 1 h in the absence or presence of an isotype control IgG2a antibody or a function-blocking monoclonal integrin α10 antibody. Bovine serum albumin (BSA)-coated wells were used as a negative and non-specific control. Adhered cells were quantified by crystal violet and spectrophotometric analysis. The bar graph shows percentage cell adhesion compared with non-treated control cells. Data are one representative experiment, and error bars show the SD in triplicates. Statistically significant differences were determined by unpaired two-tailed Student’s *t*-test, where * *p* ˂ 0.05; ** *p* ˂ 0.01; *** *p* ˂ 0.001. n.s.: not significant. (**B**) Unsorted U3054MG cells cultured as spheres. Triple immunofluorescence labeling of integrin α10 protein, Sox2 and Nestin, DAPI staining of cell nuclei, and a merged image (Merge). Scale bars represent 10 µm. (**C**) The number of integrin α10-positive cells measured by flow cytometry of α10^high^- and α10^low^-sorted U3054MG cells grown in monolayer or as spheres. Data are presented as the mean from three independent experiments. (**D**) Sphere formation capacity of α10^high^-and α10^low^-cells measured after 7 days of culturing. Microscopy images of each well were taken and spheres that had reached a diameter of more than 100 µm were counted using ImageJ software. Data are presented as the mean from five independent experiments. (**E**) Cell proliferation of α10^high^- and α10^low^- cells after 4‒5 days of culturing as spheres was determined by using the WST-1 cell proliferation assay. Data are presented as the mean from four independent experiments. The number of spheres and relative cell number is normalized against α10^low^-cells. Error bars show the SD, and the unpaired two-tailed Student’s *t*-test was performed, where * *p* ˂ 0.05; ** *p* ˂ 0.01; *** *p* ˂ 0.001.

To investigate whether integrin α10β1, which is known as a collagen receptor, can also mediate adhesion of GBM cells to laminin, GBM cells were allowed to adhere to laminin-111-coated dishes in the presence of a function-blocking antibody to integrin α10 (Figure 4A). The specificity of the antibody was demonstrated using C2C12 cells transfected with either human α10- or human α11-integrin subunits (Figure 4A). We found that integrin α10β1 can utilize laminin-111 as a ligand in addition to various collagens (Figure 4A).

#### 2.4.2. Integrin α10β1 Promotes Proliferation of GBM Cells and Sphere Formation

When we examined integrin α10 expression levels in spheres by immunofluorescence, we found that all cells express α10 but at different levels. Moreover, we also found partial cellular co-expression of integrin α10β1 with both Sox2 and Nestin in the spheres (Figure 4B). We then sorted U3054MG cells into integrin α10^high^ and α10^low^ populations and cultured the cells as spheres or in a monolayer on laminin-111 coated plates. We found that the expression of integrin α10β1 was drastically increased when the cells were cultured as spheres compared with cells grown in a monolayer, on both α10^high^- and α10^low^- U3054MG cells (Figure 4C). Integrin α10^high^-cells, however, showed significantly increased sphere formation and increased proliferation compared with integrin α10^low^cells (Figure 4D,E).

#### 2.4.3. *ITGA10* Knockdown in GBM Cells Reduces Migration and Increases Cell Death

**Figure 5:**
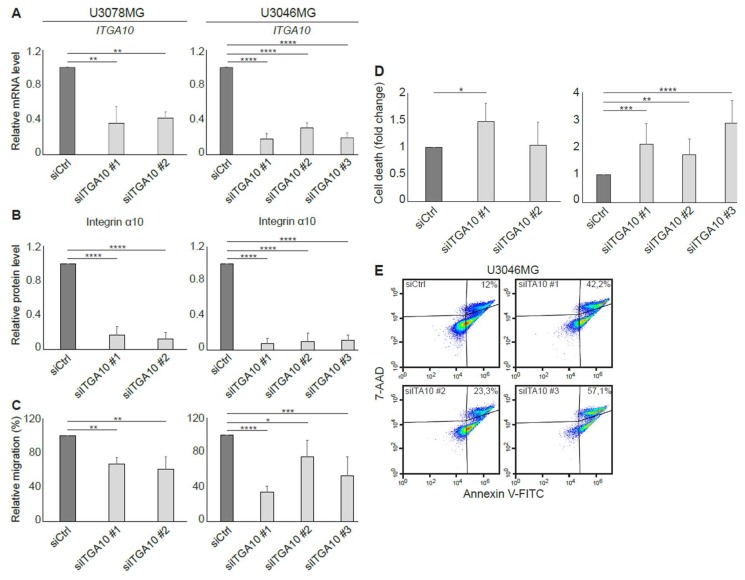
Reduced cell migration capacity and increased cell death of *ITGA10* knockdown in GBM cells. The U3078MG and U3046MG cells were transfected for 96 h with siRNA against integrin α10 (si*ITGA10*) or an unspecific siRNA (siCtrl) as control. (**A**) qRT-PCR analysis of mRNA expression of *ITGA10* in the knockdown GBM cells. Expression data were normalized to the reference gene GAPDH. Data are presented as mean ± SD from three independent experiments. (**B**) Flow cytometry analysis of integrin α10 protein levels in the GBM cells. Data are presented as mean from 8–12 individual experiments. siCtrl is set to 1 for mRNA and protein levels and error bars show the SD. (**C**) Migration capacity of siCtrl and si*ITGA10* knockdown cells, evaluated by the transwell migration assay. Data are presented as mean from 3‒6 independent experiments. siCtrl is set to 100% and error bars show the SD. (**D**) Number of dead cells evaluated by an Annexin/7-Aminoactinomycin-D (7-AAD) assay and flow cytometry. The flow cytometry and 7-AAD results are presented as the mean from 8‒12 individual experiments and the 7-AAD results demonstrate fold change compared with siCtrl. One Annexin/7-AAD experiment analyzed by FlowJo software is shown in Figure (**E**). Statistically significant differences were determined by one-way ANOVA followed by Dunnett, where * *p* ˂ 0.05; ** *p* ˂ 0.01; *** *p* ˂ 0.001; and **** *p* ˂ 0.0001.

*ITGA10* expression was knocked down in two GBM cell lines with high integrin α10 levels, U3078MG and U3046MG, using siRNA. After 96 h, a specific knockdown of *ITGA10* was demonstrated both on mRNA and the cell surface protein level (Figure 5A,B). As demonstrated in Figure 5C, we found that cell migration on gelatin-coated transwell filters was significantly diminished in *ITGA10* knockdown cells. Moreover, by knocking down *ITGA10*, the viability was reduced, seen as an increased number of dead cells (7-Aminoactinomycin D (7-AAD) positive cells) compared with the control transfected cells (Figure 5D,E). These results suggest that integrin α10β1 is important for both the migration and survival of GBM cells.

### 2.5. Targeting Integrin α10β1 In Vitro and In Vivo with an ADC

#### Integrin α10 Antibody Conjugated with Saporin Induces the Death of GBM Cells

**Figure 6:**
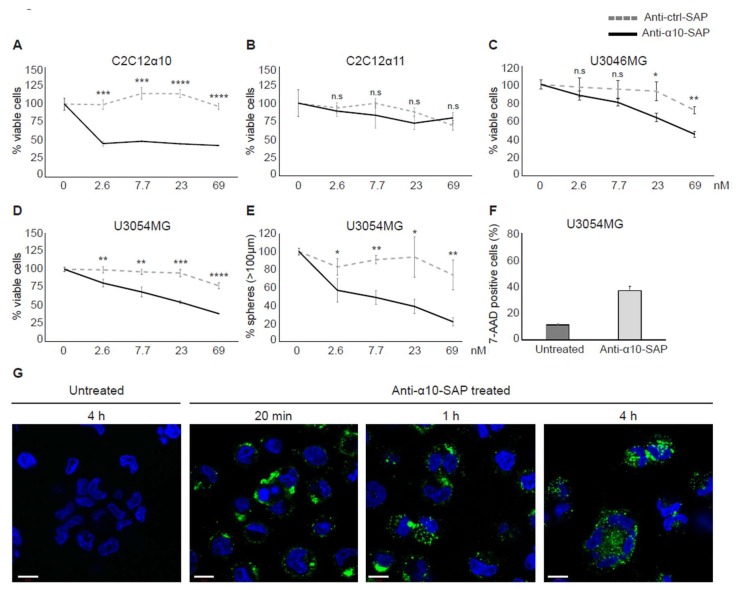
The antibody–drug conjugate (ADC), ribosome-inactivating cytotoxin saporin (anti-α10-SAP), reduces the viability of integrin α10-cells. (**A**,**B**) Viability analysis of mouse myoblast cells C2C12, transduced with integrin α10 vector (C2C12α10) or integrin α11 vector (C2C12α11) after treatment with anti-α10-SAP or an isotype control IgG antibody conjugated to saporin (anti-ctrl-SAP) for 96 h. (**C**,**D**) Viability analysis of U3046MG and U3054MG GBM cells after treatment with increasing concentration of anti-α10-SAP or anti-ctrl-SAP for 96 h. The relative amounts of viable cells were evaluated using the WST-1 assay and presented as a percentage of viable cells compared with the untreated controls. Viability data are one representative experiment per cell line, and error bars show the SD within triplicates. (**E**) Number of spheres formed by U3054MG cells after treatment with anti-α10-SAP or anti-ctrl-SAP for 7 days. Spheres bigger than 100 µm were counted. Data are one representative experiment, and error bars show the SD within triplicates. Unpaired two-tailed Student’s *t*-test were performed, where * *p* ˂ 0.05; ** *p* ˂ 0.01; *** *p* ˂ 0.001, **** *p* ˂ 0.0001. n.s.: not significant. (**F**) Flow cytometry analysis of U3054MG cells, cultured in the presence or absence of 20‒22.5 nM anti-α10-SAP for 7 days, stained with 7-AAD. Data are presented as a mean of two independent experiments, and error bars show the SD. (**G**) Binding and internalization of anti-α10-SAP visualized at 20 min, 1h, and 4 h by immunolabeling of the monoclonal integrin α10 antibody with secondary IgG antibodies against mouse IgG (conjugated with Alexa Fluor 488). Nuclei were stained blue with DAPI. Scale bars represent 10 µm.

To investigate integrin α10β1 as a potential drug target in GBM, we constructed an ADC that combines a monoclonal integrin α10 mouse antibody with the ribosome-inactivating cytotoxin saporin (anti-α10-SAP). The specificity of the anti-α10-SAP conjugate was demonstrated using C2C12 cells transduced with either integrin α10 or integrin α11 vectors. When C2C12 cells, cultured in a monolayer, were treated with anti-α10-SAP or an isotype control IgG antibody conjugated to saporin (anti-ctrl-SAP) for 96 h, the number of viable α10-expressing C2C12 cells was significantly reduced compared with those subjected to the anti-ctrl-SAP treatment (Figure 6A). In contrast, the treatment did not affect the viability of the α11-expressing C2C12 cells (Figure 6B).

When the U3046MG and U3054MG GBM cells were treated with anti-α10-SAP, the number of viable cells was strongly reduced with the increasing concentration of anti-α10-SAP (Figure 6C,D). The best effect was shown with the U3054MG cells, where viability and sphere formation capacity was decreased by 50% and 70%, respectively, compared with anti-ctrl-SAP-treated cells at a concentration of 69 nM (Figure 6D,E). The control treatment, anti-ctrl-SAP, had a minor effect on viability and sphere formation. After treatment of the spheres with 20‒22.5 nM anti-α10-SAP over a period of 7 days, the number of dead cells (7-AAD-positive cells) was strongly increased compared with the untreated cells (Figure 6F).

To verify the binding of anti-α10-SAP to U3054MG cells and subsequent internalization, the conjugate was detected by immunolabeling of the integrin α10 antibody and analyzed by confocal microscopy. After 20 min, the anti-α10-SAP ADC was detected mainly on the cell membrane, whereas intracellular detection of the anti-α10-SAP was increased after 1‒4 h of treatment (Figure 6G).

Taken together, these results demonstrate that our ADC selectively binds to the cell surface integrin α10β1 and is internalized into the cytoplasm where the delivered saporin toxin causes cell death.

### 2.6. Anti-Tumor Effects of Anti-α10-SAP in an Orthotopic Xenograft Model 

To further investigate the role of integrin α10 as a target in GBM, we investigated the effect of anti-α10-SAP in an orthotopic xenograft model established in nonobese diabetic/severe combined immunodeficiency (NOD-SCID) mice using the GBM U3054MG cells. The cells were injected intracranially, and the tumors were allowed to grow for 5 or 7‒8 weeks in the absence or presence of anti-α10-SAP or anti-ctrl-SAP administered intracerebroventricularly.

**Figure 7:**
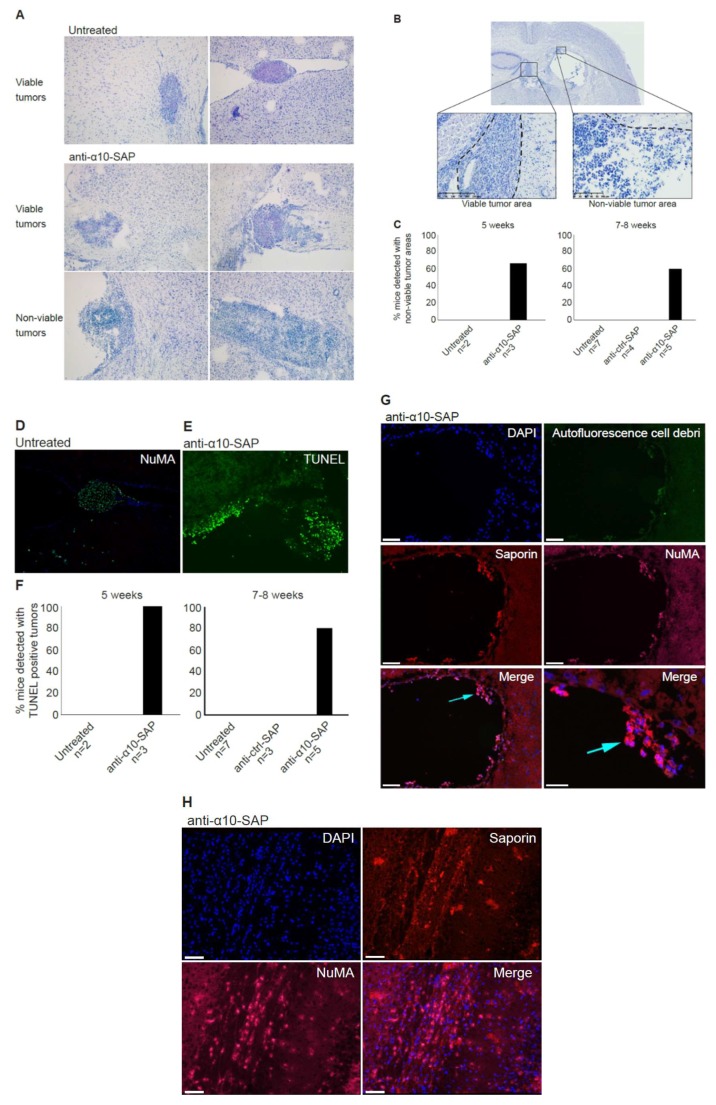
The in vivo efficacy of anti-α10-SAP treatment on GBM cells in an orthotopic xenograft model. GBM orthotopic xenografts were established in nonobese diabetic/severe combined immunodeficiency (NOD-SCID) mice via intracranial (striatum) stereotactic injection of U3054MG cells. Intracerebroventricular injection of anti-α10-SAP or anti-ctrl-SAP was performed, followed by histopathological assessment of the brains 5 and 7‒8 weeks after cell injection. (**A**) Representative toluidine blue-stained brain sections demonstrating orthotopic xenograft tumors. Small-to-large viable tumors, established in brain tissue and the ventricle, are shown in untreated and anti-α10-SAP-treated mice. In the two lower squares, examples of tumors in the ventricle with non-viable areas are shown. (**B**) Definition of viable and non-viable tumor areas: viable tumor cell formations showed typical cancerous neoplastic cells with a large and irregular shaped nucleus, prominent nucleoli, and predominantly a pale cytoplasmatic toluidine blue staining, as well as a cell population with a high grade of mitosis, including atypical mitotic forms; non-viable tumor areas showed atypical characteristics with dead or dying cells, the presence of inflammatory cells, cell membrane, and/or nuclear debris. (**C**) Percentage of mice detected with non-viable tumor areas by histopathological evaluation in untreated (*n* = 2) and anti-α10-SAP-treated (*n* = 3) mice after 5 weeks, and in untreated (*n* = 7), anti-ctrl-SAP- and anti-α10-SAP-treated (*n* = 4 and *n* = 5, respectively) groups after 7‒8 weeks. Non-viable tumor areas were found at all time points but only in the anti-α10-SAP-treated mice. (**D**) NuMA immunofluorescence labeling (green) of human GBM cells established as a tumor in the ventricle and DAPI staining of cell nuclei (blue). (**E**) Detection of dead tumor cells visualized with terminal deoxynucleotidyl transferase dUTP nick end labeling (TUNEL) fluorescence staining (green) in brain tissue after intracerebroventricular treatment with anti-α10-SAP. (**F**) The induction of cell death was evaluated by TUNEL staining. The percentage of mice with TUNEL-positive tumors in the untreated (*n* = 2) and anti-α10-SAP-treated (*n* = 3) mice after 5 weeks, and in untreated (*n* = 7), anti-ctrl-SAP-, and anti-α10-SAP-treated (*n* = 3 and *n* = 5, respectively) groups after 7‒8 weeks are shown. Immunofluorescence labeling of colocalized saporin (red) and NuMA (magenta) in tumor cells in the ventricle (**G**) and in brain tissue (**H**) after intracerebroventricular treatment of anti-α10-SAP. Autofluorescence from cytoplasmic debris from dead cells (green) can also be observed, together with nuclear debris (DAPI-stained blue). Scale bars represent 40 µm, and the bottom right represents 20 µm in (**G**) and 50 µm in (**H**).

The injected GBM cells developed tumors of variable sizes, widely spread within the brain parenchyma, third ventricle, and the subarachnoid space (Figure 7A). Viable tumors were detected in 21 of the total 26 mice included in the study. The effect of the treatment was investigated by histopathological evaluation of the brain tissue by toluidine blue staining. In mice treated with anti-α10-SAP, non-viable tumor areas composed of dying and dead tumor cells, cell debris, and inflammatory cells were identified, and the effect was already seen after 5 weeks (Figure 7A‒C). In contrast, we could not detect any non-viable tumor structures in untreated and anti-ctrl-SAP-treated mice (Figure 7A–C). 

To further investigate and verify the histopathological findings, immunofluorescence labeling of NuMA, a specific marker for human cells, was examined in adjacent sections. The NuMA immunolabeling confirmed successful in vivo engraftment and growth of U3054MG cells with a similar pattern and distribution demonstrated by toluidine blue staining. This is shown in Figure 7A (upper right panel), while Figure 7D shows toluidine blue staining and NuMA immunofluorescence labeling of the same tumor in the ventricle of an untreated mouse. Additionally, terminal deoxynucleotidyl transferase dUTP nick end labeling (TUNEL) staining was performed to confirm cell death in the histologically defined, non-viable tumor areas (Figure 7E). Positive TUNEL staining was found in all anti-α10-SAP-treated mice after 5 weeks and in 4 out of 5 mice after 7‒8 weeks, whereas no positive TUNEL staining was found in the untreated and anti-ctrl-SAP-treated mice (Figure 7F). To confirm that the anti-α10-SAP conjugate binds specifically to the tumor cells, immunofluorescence labeling of saporin and NuMA staining was performed on brain tissues from both anti-α10-SAP-treated and anti-ctrl-SAP-treated mice. We detected saporin in both the ventricle (Figure 7G) and brain tissue (Figure 7H) of the anti-α10-SAP-treated mice, whereas no saporin immunodetection was observed in the anti-ctrl-SAP-treated mice. In addition, double immunofluorescence labeling showed that saporin was mainly localized on NuMA positive tumor cells in the anti-α10-SAP treatment group (Figure 7G). Taken together, our results demonstrate that the injected anti-α10-SAP binds specifically to NuMA-positive U3054MG tumor cells, which leads to cell death and non-viable tumor areas. This supports that integrin α10β1 is a promising novel target for the treatment of GBM and other high-grade gliomas. 

## 3. Discussion

We have previously reported that integrin α10β1 is a major collagen binding integrin on chondrocytes [24]. Moreover, we have also discovered that integrin α10β1 is expressed by MSCs isolated from bone marrow, and that FGF-2 induces the expression of integrin α10β1 [26] and promotes differentiation of MSCs to chondrocytes [24,26]. More recently, we reported that a subfraction of adipose tissue-derived MSCs selected with antibodies directed to integrin α10 have improved differentiation capacity compared with unselected MSCs [27], further indicating that integrin α10β1 correlates with stemness characteristics. Because several studies have reported that GBM cells have stem cell characteristics that contribute to their resistance to different treatment strategies, we set out to investigate integrin α10β1 expression in GBM. Initial database screening for integrin α10β1 in the human protein atlas (https://www.proteinatlas.org/) revealed that *ITGA10* mRNA was highly up-regulated in the GBM cell line U138MG, which motivated further investigation. In this study, we have shown that the cell surface protein integrin α10β1 is expressed in tumor tissues from glioma patients and in patient-derived GBM cell lines. We have also demonstrated that the expression of α10β1 is extensively expressed in high-grade gliomas (astrocytoma grade III) and GBM compared with low-grade gliomas (astrocytoma grade II) where expression is more dispersed. These data are in line with previous observations that integrin α10β1 is up-regulated in malignant melanoma and distant metastasis in myxofibrosarcoma [28,30]. Moreover, we show that high *ITGA10* expression correlates with a worse overall survival probability in glioma patients and may play a critical role in tumor progression in glioma.

In this study, we used the HGCC cell lines, because these cells are more similar to the original tumor cells than serum-cultured cell lines [32]. Indeed, we found a higher expression of integrin α10β1 in these GBM cell lines under neural stem cell culture conditions compared with both serum-cultured GBM cells and conventional GBM cell lines, such as U87MG. We also found cellular colocalization of integrin α10β1 and the stemness markers Nestin and Sox2 in the majority of all tested GBM cells (Figure 3A), suggesting that integrin α10β1 is expressed on GBM cells with a stem/progenitor-like phenotype. When we examined GBM cells cultured as spheres, however, we found that GBM cells with high integrin α10β1 expression had low Nestin expression, and vice versa (Figure 4B). Additionally, immunohistochemical analysis of GBM tumor tissues demonstrated that only a few integrin α10β1-expressing GBM cells (between 0‒10% in different patient samples) were co-expressed with Nestin, and even fewer of the integrin α10β1-expressing GBM cells were co-expressed with Sox2 (Figure 3B). The discrepancy between the cells in culture and those examined in the GBM tissues may be because patient-derived GBM cells were cultured under conditions that maintain stem cell characteristics.

Our findings that cell surface expression of integrin α10β1 is increased during sphere formation compared with monolayer cultures and that integrin α10β1-selected GBM cells have an increased ability to grow as spheres indicate that integrin α10β1 has an important role in glioma growth. Integrins have an important role in the cell matrix and mediating cellular functions, such as adhesion, migration, and proliferation, although their specific role in GBM cells is unclear. A number of integrins have been demonstrated to be expressed by GBM cells [20,36] and the integrin subunits α3, α6, and α7 are not only up-regulated in glioma stem-like cells, but increase their tumorigenicity [33,34,35,37]. Because these integrin subunits are all laminin-binding receptors, we decided to investigate whether integrin α10β1, a collagen receptor [24], could also mediate the binding of GBM cells to laminin. Previous studies by Tulla et al. have demonstrated binding of the integrin α10 I domain to laminin-111 [38]; however, this has not been verified with integrin α10β1-expressing cells. Using a function-blocking antibody to integrin α10, we were able to demonstrate that integrin α10β1 can indeed mediate adhesion of GBM cells to laminin-111, indicating that integrin α10β1 on GBM cells may use laminin as a ligand in addition to collagen. It has previously been reported that the integrins α1β1 and α2β1 can both bind laminin [38]. These integrins, as well as integrins α10β1 and α11β1, are well-characterized collagen-binding integrins involved in cellular functions, such as adhesion and migration, although their biological function in vivo is not fully understood [39]. A recent publication has shown that integrin α10β1 binds to cartilage collagen molecules, but not to fibrillar collagens [40], which indicates that integrin α10β1 may interact with matrix structures other than collagen fibrils in the GBM tumors. Our results from GBM tissues have shown that integrin α10β1 expression on single cells was dispersed within the tumor, on cells in the vicinity of blood vessels, and on large cell clusters within the tumor mass. The specific integrin α10β1–matrix interaction in these different areas remains to be elucidated.

In agreement with a previous study, where integrin α10β1 was shown to be associated with melanoma cell migration [28], our results suggest that integrin α10β1 is involved in the migration of GBM cells as demonstrated using siRNA and suggested by our gene expression analysis of the integrin α10^high^-selected GBM cell population. In the microarray data, eight of the top 20 up-regulated genes in the high α10 intensity populations have previously been shown to be involved in migration and/or invasion. Furthermore, integrin α10β1 on ovarian tumor cells binds to a cryptic epitope, HU177, present within multiple forms of collagen after denaturation [29]. Blocking this interaction caused diminished migration of α10β1-expressing, fibroblast-like cells on denatured collagen, as well as reduced proliferation of ovarian tumor cells, decreased angiogenesis, and accumulation of α-SMA-expressing stromal cells [29]. Together, these novel data indicate that integrin α10β1 has an important role in cell proliferation, migration, and survival.

Our finding that integrin α10β1 is highly expressed by tumor cells in high-grade gliomas, in combination with the restricted expression of integrin α10β1 in normal tissues, suggests that it is both a novel marker and an attractive target for therapeutic intervention in GBM. We employed an ADC approach using antibodies against α10β1 to target invasive cells by specifically delivering saporin to the integrin α10β1-expressing tumor cells. Saporin is a ribosome inactivating protein (RIP) that in minute quantities can stop protein synthesis, thereby causing cell death [31]. We have shown that our ADC complex (anti-α10-SAP) binds, internalizes, and subsequently kills the targeted cells.

Today, several ADC therapies are in development for the treatment of different tumor types, including glioma [41]. The ADCs are an attractive treatment approach because of their specific and targeted delivery of a highly potent cytotoxic drug into tumor cells. Our study shows that integrin α10β1 represents a promising target for ADC treatment of GBM due to its localization at the cell surface, its involvement in important tumor cell functions, such as cell migration and viability, and its overexpression in glioma tissue and restricted expression in normal tissue. In addition, we demonstrated that our ADC complex binds specifically to integrin α10β1-expressing cells and reduces the GBM cell viability and sphere formation in a dose-dependent manner. To ensure the ADC specificity for integrin α10β1, α11β1-expressing C2C12 cells were used as a negative control, because α11 is the integrin subunit most closely related to α10 and both are collagen binding integrins. 

In the in vivo study, non-viable tumor tissue was only detected in the anti-α10-SAP-treated mice, demonstrating a targeted anti-tumor effect of anti-α10-SAP by its ability to cause specific tumor cell death. We performed a histological evaluation in combination with a TUNEL assay to demonstrate tumor cell death. The TUNEL assay confirmed the histopathological, non-viable tumor identification, but also detected additional non-viable tumors. A likely explanation is that TUNEL staining is a more sensitive method of detecting cell death compared with histopathological evaluation. 

In this study, the ADC was delivered by injection into the cerebrospinal fluid (CSF) of the ventricle instead of local delivery to the tumor injection site, because GBM is not a localized disease and can affect and recur throughout the entire brain. Thus, CSF delivery via Ommaya intracerebroventricular or lumbar puncture is a promising treatment for GBM, both upon its initial presentation and after cellular tumor spread through the CSF following tissue disruption caused by resection neurosurgery. Notably, intracerebroventricular and lumbar intrathecal injections of chemotherapy drugs, such as methotrexate and other, newer agents, into the CSF on a weekly basis by neuro-oncologists is a standard patient treatment for CNS lymphoma. To ensure extensive dissemination of anti-α10-SAP in the mouse brain tissue and that it reached tumor cells throughout the entire CNS via the CSF pathways, including the brain parenchyma via the Virchow–Robin perivascular spaces [42], we chose to inject anti-α10-SAP through a guide screw in the ventricle. Staining the mouse brain tissue with an antibody to saporin allowed us to demonstrate that the anti-α10-SAP did indeed reach the intraparenchymal brain tissue. 

Taken together, the ADC experiments, demonstrating the anti-α10-SAP-induced cell death of GBM cells both in vitro and in vivo, further support the potential of integrin α10β1 as a novel therapeutic target in GBMs.

## 4. Materials and Methods

### 4.1. Cell Lines

Glioblastoma cells from the HGCC (www.hgcc.se) resource at Uppsala University were used. The HGCC cells are derived from patient material obtained from brain tumor tissue samples collected as surgical biopsies [32]. The cells were cultured in serum-free, defined neural stem cell (NSC) medium containing Dulbecco’s Modified Eagle Medium (DMEM)/F12 w/Glutamax and Neurobasal media (1:1) (Gibco, Scotland, UK), supplemented with B27 (Gibco), N2 (Gibco), 100 U/mL Antibiotic-Antimycotic (Gibco), bFGF (10 ng/mL) (Miltenyi, Bergisch Gladbach, Germany), and EGF (10 ng/mL) (Gibco) at 37 °C with 5% CO_2_. The adherent cell lines were cultured and expanded on Corning Primaria cell culture dishes coated with laminin-111 (1:100, L2020, Sigma Aldrich, St. Louis, MO, USA). The mouse myoblast cell line C2C12 transduced with integrin α10 vector (C2C12α10) or integrin α11 vector (C2C12α11) was cultured in Dulbecco’s Modified Eagle Medium (Gibco) supplemented with 10% Fetal bovine serum (FBS, Biological Industries, Beit Haemek, Israel) and 100 U/mL Antibiotic-Antimycotic (Gibco). The C2C12α10 and C2C12α11 cells were selected using G418 (1 µg/µL) and puromycin (10 µg/mL), respectively. As part of our laboratory routine, all cell lines were regularly tested for mycoplasma and replaced on a tri-monthly basis.

### 4.2. Analysis of TCGA and HGCC Gene Expression Data

Normalized TCGA gene expression data (IlluminaHiSeq, San Diego, CA, USA) was downloaded using the UCSC Cancer Browser (genome-cancer.ucsc.edu) for low-grade glioma and GBM together with corresponding clinical information. Clinical information was used to stratify patients into astrocytoma grade II, astrocytoma grade III, and GBM. One-way ANOVA test and the non-parametric alternative (Kruskal–Wallis test) were used to test for a difference in expression between the three groups. 

Furthermore, normalized RNA-seq data for *ITGA10* together with clinical data from a curated, published dataset [43] originating from the TCGA-LGG and TCGA-GBM cohorts, was downloaded from the Gliovis website (http://gliovis.bioinfo.cnio.es/) [44]. Patients diagnosed with oligodendroglioma and oligoastrocytoma were excluded from the study; remaining patients with an assigned grade (*n* = 59, 116, and 149 patients for grades II and III and GBM, respectively) were divided into groups according to high and low *ITGA10* expression based on a median cut-off for the subsequent survival analysis by the Kaplan–Meier method and log-rank test. 

Gene expression z-scores for *ITGA10* from the 48 published HGCC cell lines were downloaded from hgcc.se. 

### 4.3. Flow Cytometry Analysis and Fluorescence-Activated Cell Sorting

Immunostaining of GBM cells was performed by incubating cells with antibodies against integrin α10 (Alexa Fluor 647 conjugate), α3, α6, and α7 (see Table S2) for 30 min in the dark at 4 °C prior to flow cytometry analysis using a BD Accuri C6 flow cytometer. After a 30-min incubation with a primary antibody, cells were washed twice with Dulbecco’s phosphate-buffered saline (DPBS, SH3002802, Hyclone, Logan, UT, USA) containing 1% FBS and 0.1% sodium azide and incubated with a secondary antibody (Table S3) for 20 min in the dark at 4 °C. For co-staining integrin α3 and α7 with integrin α10, cells were then incubated with integrin α10 Alexa Fluor 647 antibody for 30 min in the dark at 4 °C before subsequent analysis using flow cytometry. 

GBM cells with high expression of integrin α10β1 (α10^high^) or low expression of integrin α10β1 (α10^low^) were sorted by fluorescence-activated cell sorting (FACSAria, BD Biosciences, San Jose, CA, USA). Discrimination of live/dead cells was accompanied by 7-AAD staining (BioLegend, San diego, CA, USA). Sorted cells were washed in medium and re-seeded for recovery and expansion, and some were immediately frozen for RNA extraction. 

### 4.4. Gene Expression Microarray Analysis in Sorted Cells

The RNA concentration and purity were determined using Nanodrop and Bioanalyzer. Total gene expression analysis was performed by SCIBLU Genomics at Lund University using Human Gene 2.0 ST arrays. Basic Affymetrix chip and experimental quality analysis were performed using Expression Console Software v1.1.2 (Thermo Fisher Scientific, Santa Clara, CA, USA). Probe summarization and data normalization were done by robust multi-array analysis (RMA) [45]. Probe sets without signal intensity above the median of negative control intensity signals in at least 80% of samples were excluded, and controls and probe-sets with no gene symbol were filtered. Signals were log 2-transformed. A linear model was applied to adjust for the paired sample design using the limma package in R. The fitted values were used for the empirical Bayes method to calculate the *t*-statistics. Genes were sorted by fold change after excluding those that were not expressed differently in all three cell lines, and a *p*-value > 0.05 was used to estimate the top, differentially expressed genes.

### 4.5. RNA Extraction

Total RNA was extracted using QIAzol lysis reagent (Qiagen) for the samples used in the microarray analysis and for qRT-PCR. Total RNA was purified with a RNeasy Plus Mini kit (Qiagen, Hilden, Germany) according to the manufacturer´s instructions. The cDNA was synthesized with SuperScript VILO (Invitrogen, Carlsbad, CA, USA) and then amplified using qRT-PCR for evaluation of relative mRNA expression levels in the Applied Biosystems StepOne Plus Real Time PCR System device using the TaqMan Universal Master Mix II (Applied Biosystems, Carlsbad, CA, USA). The comparative Ct method was used to quantify relative mRNA expression, and *GAPDH* was used as a reference gene; each reaction was performed in duplicate. The TaqMan primers are listed in Table S4.

### 4.6. siRNA Transfections

The U3078MG and U3046MG GBM cells were seeded in antibiotic-free medium in 60 mm plates 24 h before transfection. Thereafter, cells were transfected for 96 h using Lipofectamine RNAiMAX (Invitrogen) and 2.25 pmol siRNA in Opti-MEM (Gibco) according to the supplier’s protocol. Then, 5 h after transfection, 15 ng/mL bFGF2 was additionally added to the plates. Non-targeting siRNA (ThermoFisher Scientific, Carlsbad, CA, USA) was used as a negative control. The oligonucleotides used were silencer select control no. 2 (4390846; ThermoFisher Scientific) and three different silencer select *ITGA10* oligos (s16180, s16181, and s16182; ThermoFisher Scientific).

### 4.7. Antibody–Saporin Conjugation

Conjugation of saporin to the monoclonal mouse integrin α10 antibody (Xintela AB, Lund, Sweden) to generate anti-α10-SAP was performed by Advanced Targeting Systems (San Diego, CA, USA). As a control for the anti-α10-SAP targeting, we used two different anti-ctrl-SAP preparations (Advanced Targeting Systems and Innovagen AB, Lund, Sweden).

### 4.8. Migration Assay

The trans-well inserts (CytoSelect Cell Migration Assay kit, Cell Biolabs, San Diego, CA, USA) were coated with gelatin (Sigma Aldrich, Saint Louis, MO, USA) for approximately 2‒4 h at 37 °C. The *ITGA10*-knocked-down U3078MG and U3046MG cells were seeded to the upper compartment of the CytoSelect Insert. The lower compartment was filled with NSC medium supplemented with 5% FBS. After incubation for 72 h at 37 °C, the inserts were collected, and the cells adhering to the lower surface were fixed, stained with the cell stain solution, and the extraction was quantified by measuring the absorbance at 560 nm in a SpectraMax i3 multi-mode plate reader (Molecular Devices, San Jose, CA, USA) according to the manufacturer’s recommendation.

### 4.9. Sphere Formation, Cell Proliferation, and Apoptosis Assay 

The U3054MG GBM cells were seeded at a density of 2500 cells/well in an uncoated 96-well plate, and treated with anti-ctrl-SAP and anti-α10-SAP antibody for 7 d. Whole microscopy images of each well were taken and spheres that had reached a diameter of 100 µm were counted in the digital images using ImageJ software (ImageJ 1.51k, LOCI, University of Wisconsin, Madison, WI, USA) [46]. 

The cell viability was determined by using the WST-1 assay (Roche, Mannheim, Germany) according to the manufacturer´s instructions. All conditions were done in triplicate. 

To analyze apoptosis, cells were stained with the Annexin V-FITC apoptosis detection kit with 7-AAD (BioLegend, San Diego, CA, USA) according to the supplier’s protocol, and the amount of bound Annexin V-FITC and 7-AAD was quantified with a BD Accuri C6 flow cytometer. 

### 4.10. Cell Adhesion

First, 48-well dishes were coated with 0.1% gelatin (G1393, Sigma Aldrich), 10 µg/mL collagen type I from rat tail (C7661, Sigma Aldrich), 10 µg/mL collagen type II from chicken sternal cartilage (C9301, Sigma Aldrich), and 1 mg/mL laminin-111 (L2020, Sigma Aldrich) solution and blocked with 0.25% BSA (Sigma Aldrich) in PBS. BSA-coated wells were used as a negative and non-specific control. The wells were rinsed with PBS before the experiment. Cells were suspended in PBS (+Ca/Mg, Gibco) and incubated with antibodies for 30 min before plating the cells. The isotype control IgG2a antibody (401504, Biolegend) and blocking monoclonal integrin α10 antibody were used at a concentration of 3‒5 µg/mL. The C2C12α10 and C2C12α11 cells were added to the wells at a concentration of 65,000/well and U3054MG cells at a concentration of 100,000/well, and cells were allowed to adhere for 1 h at 37 °C. After incubation, cells were gently rinsed with PBS (+Ca/Mg) to remove non-adherent cells. Adherent cells were fixed with ethanol, stained with 0.09% crystal violet (Sigma Aldrich), and the absorbed dye was extracted by 10% acetic acid (Sigma Aldrich). The extraction was quantified by measuring the absorbance at 560 nm in the SpectraMax i3 multi-mode plate reader (Molecular Devices). 

### 4.11. In Vivo Study

Six-week-old female, NOD-SCID mice (NOD.CB17/AlhnRj-Prkdcscid, Janvier Labs, Le Genest St Isle, France) were housed in a controlled environment, and all procedures were carried out in accordance with an ethical permit approved by the regional ethical committee for animal research (approval no. C41/14). Mice were inoculated with 5 × 10^5^ U3054MG cells intracranially (into the striatum, injection site: AP 0, ML 1.5 (R), DV −2.5 (cells); AP 0.1, ML 0.8 (R), DV −4 (ICV)) and the tumor cells were allowed to grow in either the absence (*n* = 11) or presence of anti-α10-SAP (*n* = 11), or the isotype control ADC anti-ctrl-SAP (*n* = 8). The ADCs were first administered 1 week after cell injection. For administration, guide screws (Guide Screw 1.6 mm length 26 GA, Bilaney Consultants GmbH, Düsseldorf, Germany) were stereotactically implanted, and infusions of anti-α10-SAP and anti-ctrl-SAP were made intracerebroventricularly. Three untreated and three anti-α10-SAP treated mice were sacrificed 5 weeks after cell injection to investigate the potential effect at an early time point. The mice sacrificed after 5 weeks had received treatments once per week for 5 weeks, and the other mice had received one additional treatment (1 µg in 2 µL per infusion). The mice were monitored at least every second day. The brain tissues were snap-frozen and cryo-sectioned for further analysis.

In total, 30 mice were utilized for this study; however, four mice were excluded in the analysis. One mouse in the untreated group had to be euthanized after cell injection due to weight loss following surgery, and one mouse in the anti-ctrl-SAP group was unable to wake up after blood sampling at week 4. In the anti-α10-SAP group, two mice were excluded because of technical failure during animal preparation. 

### 4.12. Immunolabelings of Human and Mouse Brain Tissue and GBM Cell Lines

#### 4.12.1. Immunohistochemistry

Human brain tissues were obtained from archival neurosurgical tissue biopsies embedded in paraffin blocks (Lund University). Ethical permission was obtained from the Regional Ethics Committee at Lund University (Dnr 2019-00598). Tissue samples contained regions with both normal tissue and tumor histopathology, as determined by a clinical pathologist (E.E.). Tissues were sectioned at 5 µm and collected on microscope slides (SuperFrost plus, Thermo Scientific). Sections were then re-hydrated and de-paraffinized by immersion in xylene (100% × 2) followed by immersion in a graded alcohol series (100% ethanol 1 min × 2, 95% ethanol 1 min, 70% ethanol 1 min × 2), ending with distilled water. Heat-induced antigen retrieval was performed in citrate buffer, pH 6.0 (10 mM sodium citrate) containing 0.05% Tween 20 (Sigma-Aldrich, St. Louis, MO, USA) for 10 min at 90 °C, followed by immersion in distilled water for 10 min and in PBS (3 × 3 min). Sections were then incubated in PBS containing 0.3% H_2_O_2_ for 10 min, followed by rinses in PBS (3 × 3 min). Sections were incubated in PBS containing 0.05% Triton X-100 (AppliChem, Darmstadt, GmbH) and 1% bovine serum albumin (BSA; Sigma-Aldrich, PBS-TX-BSA) for 30 min at room temperature (RT). Incubation was performed in primary antibodies made in rabbit against α10 (Xintela AB, diluted in PBS-TX-BSA) for 16 h at 4 °C. After rinses in PBS (3 × 3 min), sections were incubated with horseradish peroxidas (HRP)-conjugated secondary antibodies (goat anti-rabbit, DAKO Envision-HRP, Denmark) for 45 min at RT. Following rinses in PBS (3 × 3 min), sections were incubated for 10 min in PBS containing 3,3′-di-aminobenzidine (DAB, 25 mg/mL) and 0.05% H_2_O_2_. Sections were then rinsed in PBS (3 × 3 min) and counterstained with hematoxylin (Mayers, Histolab Gothenburg, Sweden). Sections were then dehydrated in a graded alcohol series (70%, 96% and 100%) ending with xylene (100%). Sections were mounted in Pertex (Histolab Gothenburg, Sweden) and cover-slipped. The grading of integrin α10 expression in brain tissues from patients with different clinical diagnoses was performed by scoring labeling intensities for integrin α10 immunoreactive cells. The labeling intensity was scored as 0 (negative), 1 (weak), 2 (moderate), and 3 (strong).

#### 4.12.2. Immunofluorescence and Confocal Microscopy

Immunofluorescence labeling, including multiple labeling for simultaneous visualization of epitopes, was performed in cryosections from human and mouse tissues, and in GBM cell lines. 

The U3071MG, U3078MG, U3046MG, U3054MG, and U3073MG cells were seeded on 8-well, glass bottom, microscope chamber slides (ibiTreat, Ibidi, Germany). For single and multi-labeling, cells were briefly rinsed once in cold PBS directly followed by fixation in cold 4% paraformaldehyde (Fisher Scientific, Loughborough, UK) for 10 min. Cells were washed and permeabilized via incubation in PBS containing 0.05% Triton X-100 (AppliChem, Darmstadt, Germany) for 2 × 5 min at RT and then incubated in PBS-TX-BSA at RT for 20 min. Cells were incubated with primary mouse antibodies made against integrin α10 (Xintela AB) either alone or together with antibodies against investigated epitopes (made in other species, see Table S2), for 90 min at RT. Following rinses in PBS, cells were incubated with a mixture of fluorophore-conjugated species-specific secondary antibodies (Table S3). Following rinses in PBS, cells were incubated in 4′,6-diamidino-2-phenylindole dihydrochloride (DAPI, Molecular Probes, Invitrogen, 0.1 µM in PBS)) solution (Molecular Probes, Invitrogen) for 10 min. 

Fresh frozen human brain tissues were embedded and frozen in optimal cutting temperature (OCT) compound (at approx. −60 °C). Frozen tissue was sectioned at 8 or 10 µm thickness and collected on SuperFrost slides (Thermo Scientific). Slides were air-dried at 37 °C for 20 min, rinsed in PBS and post-fixed in either acetone (100%) for 5‒10 min at −20 °C or paraformaldehyde (4% in PBS at 4‒8 °C, indicated optimal for NuMA labeling) for 20 min. Following rinses in PBS, sections were incubated in PBS-TX-BSA for 30 min at RT. Sections were incubated with primary rabbit antibodies made against integrin α10 (Xintela AB) alone or together with antibodies against investigated epitopes made in other species (See Table S2). For the fluorescence multilabeling, sections were incubated with a mixture of primary antibodies (diluted in PBS-TX-BSA) for 16 h at 4 °C. Following rinses in PBS, sections were incubated with fluorophore-conjugated secondary antibodies (see Table S3) for 45 min at RT. Following rinses in PBS-TX and once in PBS, sections were incubated in DAPI solution for 15 min, rinsed in PBS, and mounted in “anti-fade solution” ProLong Gold (Invitrogen, USA). 

Immunofluorescence analysis and cell and tissue imaging were performed in a laser confocal scanning system (LSM 800 or 710, Zeiss, Germany) equipped for specific detection of the emission wavelengths for the used fluorophores. The pinhole aperture was set at one airy unit or was optimized for simultaneous detection of all visualized channels in multilabelings. The laser power and detection levels were optimized for each channel. Sequential scanning and detection of individual fluorophores was performed at magnifications provided by ×40 or ×63 magnification oil immersion lenses. For illustrations, one optical section was obtained for each fluorophore (0.5‒1 µm thick depending on the lens used), and image acquisition was performed through as many cells as possible in the scanned region. For analyses of the cellular relationship between labeled epitopes (i.e., co-labeling or not), individual channels of the imaged fluorophores were merged using the LSM software. Brightness and contrast were optimized for representative illustrations.

### 4.13. Histochemistry and Histopathological Evaluation of Mouse Brains

Mouse brain cryosections (12 µm) collected on microscope slides (SuperFrost plus, Thermo Scientific) were air-dried at RT for 30 min and then stained with 0.5% toluidine blue (Sigma Aldrich, cat. no. 198161) for 2 min. The sections were dehydrated stepwise with 70% ethanol for 30 sec, 96% ethanol for 20 sec and two changes of 100% ethanol for 20 sec and 10 sec, respectively, followed by two changes in xylene (100%) for 3 min each. Labeled sections were analyzed in a bright-field transmission microscope (Leica DMRE, Germany) and in images from whole scanned sections (40× magnification, Hamamatsu Nanozoomer 2.0ht, C900-12, Hamamatsu, Japan).

In scanned images, areas with findings of tumors consisting of 10 or more tumor cells with or without signs of necrosis, borders were delineated and individually marked in the images (Hamamatsu NPD.view 2.6.13 software, Hamamatsu City, Japan). Assessment and scoring of findings were performed by two blinded experts in histology/histopathology (B.H./J.F.). Areas defined as tumor areas without signs of apoptosis or necrosis (see below) were morphologically defined by the following: (1) typical cancerous neoplastic cells with a large and irregular shaped nucleus, prominent nucleoli and predominantly with a pale cytoplasmatic toluidine blue staining; or (2) cell populations with a high grade of mitosis, including atypical mitotic forms. Tumor areas with signs of apoptosis/necrosis (non-viable tumor areas), indicating treatment effects, were defined by the following morphological criteria: tumor areas (as above) containing inflammatory cells, cell membrane and/or nuclear debris.

### 4.14. Immunofluorescence Labelings of Mouse Brains

For immunofluorescence labelings of saporin and NuMA, mouse brain cryosections (12 µm), collected on microscope slides (SuperFrost plus, Thermo Scientific) were air-dried at RT for 30 min. Sections were then post-fixed in either acetone (100%, at −20 °C for 10 min) or paraformaldehyde (4%, at RT for 20 min). Sections were rinsed in PBS and incubated with primary antibodies against saporin and/or NUMA (see Table S2) for 16 h at 4 °C. Sections were incubated with fluorescens conjugated secondary antibodies and processed further as described above (for human frozen tissues). Analyses and imaging were performed in a wide-field epifluorescence microscope (Olympus IX73, Tokyo, Japan). Illustrations were adjusted for brightness and contrast.

For labeling of apoptotic and/or dead cells, terminal deoxynucleotidyl transferase dUTP nick end labeling (TUNEL) was performed according to the process recommended by the manufacturer (Roche, cat no 11 684 795 910). All labelling experiments included negative control sections (by excluding terminal transferase from the protocol) and positive control sections (pretreatment of sections with DNAse I). Nuclear labeling was achieved by mounting in Fluoroshield mounting media containing DAPI (Abcam, Cambridge, UK). Labeling was analyzed and documented in an epi-fluorescence microscope (Leica DMRE, Germany) and evaluated for the presence or absence of a signal (detected in the 489 nm/FITC channel) in discernable tumor areas (using nuclear morphology visualized in the 405 nm/DAPI channel). Signal presence or absence was documented as positive or negative, respectively. Brain tissue from one of the mice in the anti-ctrl-SAP-treated group was not TUNEL stained due to a lack of sufficient tissue sections. 

### 4.15. Anti-α10-SAP Targeting Analysis

Cells were fixed in cold 4% paraformaldehyde, followed by rinsing in PBS (2 × 2 min) and incubation in PBS-BSA-TX (15 min at RT). Anti-α10-SAP distribution was determined by labeling the integrin α10 antibody (made in mouse) with a secondary antibody against mouse IgG (mAb α10) conjugated with Alexa Fluor 488 (Jackson ImmunoResearch Inc., USA). Incubation of cells was performed at dilutions of 1:150‒200 for 30 min at RT. After 2 × 5 min rinses in PBS, cells were incubated in DAPI solution (Molecular Probes, Invitrogen) for 10 min. Analysis and digital imaging documentation were performed in a confocal laser scanning system (Zeiss LSM 800 or 710, as described above).

### 4.16. Statistical Analyses

Statistical analyses were performed using unpaired two-tailed Student *t*-test, as well as ANOVA followed by Dunnett, as stated in the figure legends. Four levels of significance were used, where * *p* < 0.05, ** *p* < 0.01, *** *p* < 0.001, and *p* < 0.0001.

## 5. Conclusions

In this study, we investigated the potential of integrin α10β1 as a therapeutic target in glioblastomas. Our results demonstrated that integrin α10β1 has a crucial role in the migration, proliferation, and survival of GBM cells and that an integrin α10 antibody–drug conjugate induced cell death of GBM cells both in vitro and in vivo. Our findings open up for new strategies to both diagnose and treat this aggressive and invasive form of brain cancer as well as other high-grade gliomas.

## Supplementary Materials

The following are available online at https://www.mdpi.com/2072-6694/11/4/587/s1, Table S1: The 8 genes involved in migration and/or invasion among the top 20 genes up-regulated, Table S2: The table showing the relevant information pertaining to the primary antibodies used in the context of flow cytometry and immunolabeling, Table S3: List of secondary antibodies used in the context of flow cytometry and immunolabeling, Table S4. TaqMan primers used in the qPCR analysis.

## References

[1] Zhang X., Zhang W., Cao W.D., Cheng G., Zhang Y.Q. (2012). Glioblastoma multiforme: Molecular characterization and current treatment strategy (Review). Exp. Ther. Med..

[2] Louis D.N., Perry A., Reifenberger G., von Deimling A., Figarella-Branger D., Cavenee W.K., Ohgaki H., Wiestler O.D., Kleihues P., Ellison D.W. (2016). The 2016 World Health Organization Classification of Tumors of the Central Nervous System: a summary. Acta Neuropathol..

[3] Van Meir E.G., Hadjipanayis C.G., Norden A.D., Shu H.K., Wen P.Y., Olson J.J. (2010). Exciting new advances in neuro-oncology: the avenue to a cure for malignant glioma. Cancer J. Clin..

[4] Brennan C.W., Verhaak R.G., McKenna A., Campos B., Noushmehr H., Salama S.R., Zheng S., Chakravarty D., Sanborn J.Z., Berman S.H. (2013). The somatic genomic landscape of glioblastoma. Cell.

[5] Huse J.T., Holland E.C. (2010). Targeting brain cancer: advances in the molecular pathology of malignant glioma and medulloblastoma. Nat. Rev. Cancer.

[6] Inda M.M., Bonavia R., Seoane J. (2014). Glioblastoma multiforme: a look inside its heterogeneous nature. Cancers.

[7] Patel A.P., Tirosh I., Trombetta J.J., Shalek A.K., Gillespie S.M., Wakimoto H., Cahill D.P., Nahed B.V., Curry W.T., Martuza R.L. (2014). Single-cell RNA-seq highlights intratumoral heterogeneity in primary glioblastoma. Science.

[8] Sottoriva A., Spiteri I., Piccirillo S.G., Touloumis A., Collins V.P., Marioni J.C., Curtis C., Watts C., Tavare S. (2013). Intratumor heterogeneity in human glioblastoma reflects cancer evolutionary dynamics. Proc. Natl. Acad. Sci. USA.

[9] Kreso A., Dick J.E. (2014). Evolution of the cancer stem cell model. Cell Stem Cell.

[10] Peacock C.D., Watkins D.N. (2008). Cancer stem cells and the ontogeny of lung cancer. J. Clin. Oncol..

[11] Sakariassen P.O., Immervoll H., Chekenya M. (2007). Cancer stem cells as mediators of treatment resistance in brain tumors: status and controversies. Neoplasia.

[12] Huang E.H., Wicha M.S. (2008). Colon cancer stem cells: implications for prevention and therapy. Trends Mol. Med..

[13] Singh S.K., Clarke I.D., Terasaki M., Bonn V.E., Hawkins C., Squire J., Dirks P.B. (2003). Identification of a cancer stem cell in human brain tumors. Cancer Res..

[14] Maitland N.J., Collins A.T. (2008). Prostate cancer stem cells: a new target for therapy. J. Clin. Oncol..

[15] Sale A., Berardi N., Maffei L. (2014). Environment and brain plasticity: towards an endogenous pharmacotherapy. Physiol. Rev..

[16] Neradil J., Veselska R. (2015). Nestin as a marker of cancer stem cells. Cancer Sci..

[17] Berezovsky A.D., Poisson L.M., Cherba D., Webb C.P., Transou A.D., Lemke N.W., Hong X., Hasselbach L.A., Irtenkauf S.M., Mikkelsen T. (2014). Sox2 promotes malignancy in glioblastoma by regulating plasticity and astrocytic differentiation. Neoplasia.

[18] Seymour T., Nowak A., Kakulas F. (2015). Targeting Aggressive Cancer Stem Cells in Glioblastoma. Front. Oncol..

[19] de la Rocha A.M., Sampron N., Alonso M.M., Matheu A. (2014). Role of SOX family of transcription factors in central nervous system tumors. Am. J. Cancer Res..

[20] Malric L., Monferran S., Gilhodes J., Boyrie S., Dahan P., Skuli N., Sesen J., Filleron T., Kowalski-Chauvel A., Cohen-Jonathan Moyal E. (2017). Interest of integrins targeting in glioblastoma according to tumor heterogeneity and cancer stem cell paradigm: an update. Oncotarget.

[21] Moreno-Layseca P., Streuli C.H. (2014). Signalling pathways linking integrins with cell cycle progression. Matrix Biol..

[22] Camper L., Hellman U., Lundgren-Akerlund E. (1998). Isolation, cloning, and sequence analysis of the integrin subunit alpha10, a beta1-associated collagen binding integrin expressed on chondrocytes. J. Biol. Chem..

[23] Camper L., Holmvall K., Wangnerud C., Aszodi A., Lundgren-Akerlund E. (2001). Distribution of the collagen-binding integrin alpha10beta1 during mouse development. Cell Tissue Res..

[24] Gullberg D.E., Lundgren-Akerlund E. (2002). Collagen-binding I domain integrins--what do they do?. Prog. Histochem. Cytochem..

[25] Bengtsson T., Aszodi A., Nicolae C., Hunziker E.B., Lundgren-Akerlund E., Fassler R. (2005). Loss of alpha10beta1 integrin expression leads to moderate dysfunction of growth plate chondrocytes. J. Cell Sci..

[26] Varas L., Ohlsson L.B., Honeth G., Olsson A., Bengtsson T., Wiberg C., Bockermann R., Jarnum S., Richter J., Pennington D. (2007). Alpha10 integrin expression is up-regulated on fibroblast growth factor-2-treated mesenchymal stem cells with improved chondrogenic differentiation potential. Stem Cells Dev..

[27] Uvebrant K., Reimer Rasmusson L., Talts J., Alberton P., Aszodi A., Lundgren-Akerlund E. (2019). Integrin α10β1-selected Equine MSCs have Improved Chondrogenic Differentiation, Immunomodulatory and Cartilage Adhesion Capacity. Ann. Stem Cell Res..

[28] Wenke A.K., Kjellman C., Lundgren-Akerlund E., Uhlmann C., Haass N.K., Herlyn M., Bosserhoff A.K. (2007). Expression of integrin alpha10 is induced in malignant melanoma. Cell Oncol..

[29] Caron J.M., Ames J.J., Contois L., Liebes L., Friesel R., Muggia F., Vary C.P., Oxburgh L., Brooks P.C. (2016). Inhibition of Ovarian Tumor Growth by Targeting the HU177 Cryptic Collagen Epitope. Am. J. Pathol..

[30] Okada T., Lee A.Y., Qin L.X., Agaram N., Mimae T., Shen Y., O’Connor R., Lopez-Lago M.A., Craig A., Miller M.L. (2016). Integrin-alpha10 Dependency Identifies RAC and RICTOR as Therapeutic Targets in High-Grade Myxofibrosarcoma. Cancer Discov..

[31] Polito L., Bortolotti M., Mercatelli D., Battelli M.G., Bolognesi A. (2013). Saporin-S6: a useful tool in cancer therapy. Toxins.

[32] Xie Y., Bergstrom T., Jiang Y., Johansson P., Marinescu V.D., Lindberg N., Segerman A., Wicher G., Niklasson M., Baskaran S. (2015). The Human Glioblastoma Cell Culture Resource: Validated Cell Models Representing All Molecular Subtypes. EBioMedicine.

[33] Nakada M., Nambu E., Furuyama N., Yoshida Y., Takino T., Hayashi Y., Sato H., Sai Y., Tsuji T., Miyamoto K.I. (2013). Integrin alpha3 is overexpressed in glioma stem-like cells and promotes invasion. Br. J. Cancer.

[34] Haas T.L., Sciuto M.R., Brunetto L., Valvo C., Signore M., Fiori M.E., di Martino S., Giannetti S., Morgante L., Boe A. (2017). Integrin alpha7 Is a Functional Marker and Potential Therapeutic Target in Glioblastoma. Cell Stem Cell.

[35] Lathia J.D., Gallagher J., Heddleston J.M., Wang J., Eyler C.E., Macswords J., Wu Q., Vasanji A., McLendon R.E., Hjelmeland A.B. (2010). Integrin alpha 6 regulates glioblastoma stem cells. Cell Stem Cell.

[36] Schittenhelm J., Tabatabai G., Sipos B. (2016). The role of integrins in primary and secondary brain tumors. Histol Histopathol.

[37] Lathia J.D., Mack S.C., Mulkearns-Hubert E.E., Valentim C.L., Rich J.N. (2015). Cancer stem cells in glioblastoma. Genes Dev..

[38] Tulla M., Pentikainen O.T., Viitasalo T., Kapyla J., Impola U., Nykvist P., Nissinen L., Johnson M.S., Heino J. (2001). Selective binding of collagen subtypes by integrin alpha 1I, alpha 2I, and alpha 10I domains. J. Biol. Chem..

[39] Zeltz C., Gullberg D. (2016). The integrin-collagen connection--a glue for tissue repair?. J. Cell Sci..

[40] Woltersdorf C., Bonk M., Leitinger B., Huhtala M., Kapyla J., Heino J., Gil Girol C., Niland S., Eble J.A., Bruckner P. (2017). The binding capacity of alpha1beta1-, alpha2beta1- and alpha10beta1-integrins depends on non-collagenous surface macromolecules rather than the collagens in cartilage fibrils. Matrix Biol..

[41] Gan H.K., van den Bent M., Lassman A.B., Reardon D.A., Scott A.M. (2017). Antibody-drug conjugates in glioblastoma therapy: the right drugs to the right cells. Nat. Rev. Clin. Oncol..

[42] Matsumae M., Sato O., Hirayama A., Hayashi N., Takizawa K., Atsumi H., Sorimachi T. (2016). Research into the Physiology of Cerebrospinal Fluid Reaches a New Horizon: Intimate Exchange between Cerebrospinal Fluid and Interstitial Fluid May Contribute to Maintenance of Homeostasis in the Central Nervous System. Neurol Med. Chir..

[43] Ceccarelli M., Barthel F.P., Malta T.M., Sabedot T.S., Salama S.R., Murray B.A., Morozova O., Newton Y., Radenbaugh A., Pagnotta S.M. (2016). Molecular Profiling Reveals Biologically Discrete Subsets and Pathways of Progression in Diffuse Glioma. Cell.

[44] Bowman R.L., Wang Q., Carro A., Verhaak R.G., Squatrito M. (2017). GlioVis data portal for visualization and analysis of brain tumor expression datasets. Neuro. Oncol..

[45] Irizarry R.A., Hobbs B., Collin F., Beazer-Barclay Y.D., Antonellis K.J., Scherf U., Speed T.P. (2003). Exploration, normalization, and summaries of high density oligonucleotide array probe level data. Biostatistics.

[46] Schneider C.A., Rasband W.S., Eliceiri K.W. (2012). NIH Image to ImageJ: 25 years of image analysis. Nat. Methods.

