# Supplementary material for: Integrin α10, a Novel Therapeutic Target in Glioblastoma, Regulates Cell Migration, Proliferation, and Survival
Source: Cancers (Basel). 2019 Apr 25;11(4):587. doi: 10.3390/cancers11040587 (PMC6521287; doi:10.3390/cancers11040587)
Supplement: Supplementary file 1 [file cancers-11-00587-s001.pdf]

Supplementary Materials: Integrin α10, a Novel Therapeutic Target in Glioblastoma, Regulates Cell Migration, Proliferation, and Survival

Matilda Munksgaard Thorén, Katarzyna Chmielarska Masoumi, Cecilia Krona, Xiaoli Huang, Soumi Kundu, Linnéa Schmidt, Karin Forsberg-Nilsson, Marcus Floyd Keep, Elisabet Englund, Sven Nelander, Bo Holmqvist and Evy Lundgren-Åkerlund

Table S1. The 8 genes involved in migration and/or invasion among the top 20 genes up-regulated in the $\alpha10^{high}$ expressing U3071MG, U3078MG and U3054MG cells according to microarray analysis. Log fold changes and *p*-values are shown.

No.	Gene Symbol	Gene Description	Log Fold sChange	*p*-Value	Ref.
2	CAPN6	calpain 6	1.55	0.023	[1]
4	MGP	matrix Gla protein	1.19	0.012	[2,3]
10	PDGFD	platelet derived growth factor D	1.00	0.002	[4]
11	ANGPT1	angiopoietin 1	0.97	0.0004	[5]
12	TRPV4	transient receptor potential cation channel, subfamily V, member 4	0.96	0.001	[6–8]
15	SPP1	secreted phosphoprotein 1	0.86	0.022	[9]
16	SERPINI1	serpin peptidase inhibitor, clade I (neuroserpin), member 1	0.84	0.009	[10]
18	SCIN	scinderin	0.82	0.027	[11]

Table S2. The table showing the relevant information pertaining to the primary antibodies used in the context of flow cytometry and immunolabeling.

Epitope	Conjugate	Manufacturer	Species; Monoclonal or Polyclonal
Integrin α3		LS-B13291, LSBio	Mouse; monoclonal
Integrin α6		MAB1378, Millipore	Rat; monoclonal
Integrin α7		LS-B11231, LSBio	Mouse; monoclonal
Integrin α10	Alexa Fluor 647	Xintela AB	Mouse; monoclonal
Integrin α10	PE	Xintela AB	Mouse; monoclonal
Integrin α10		Xintela AB	Rabbit; polyclonal
GFAP		ab53554, Abcam	Goat; polyclonal
Nestin		MAB5326 Chemicon®, Merck Millipore	Mouse; monoclonal
NG2		ab20156, Abcam	Mouse; monoclonal
NuMA		ab97585, Abcam	Rabbit; polyclonal
Saporin		ab18791, Abcam	Goat; polyclonal
Sox2		ab110145, Abcam	Goat; polyclonal

Table S3. List of secondary antibodies used in the context of flow cytometry and immunolabeling.

Secondary Antibodies	Manufacturer
Rhodamine Red™-X-AffiniPure F(ab′)2 Fragment Donkey Anti-Rat IgG (H+L) (cat.no. 711-296-153)	Jackson ImmunoResearch Inc. (West Grove, PA, USA)
Rhodamine Red™-X (RRX) AffiniPure F(ab′)$_2$ Fragment Donkey Anti-Rabbit IgG (H+L) (cat.no. 711-296-152)	Jackson ImmunoResearch Inc. (West Grove, PA, USA)
Alexa Fluor® 488 AffiniPure F(ab′)$_2$ Fragment Donkey Anti-Mouse IgG (H+L) (cat.no. 715-546-150)	Jackson ImmunoResearch Inc. (West Grove, PA, USA)
Alexa Fluor® 647 AffiniPure F(ab′)$_2$ Fragment Donkey Anti-Mouse IgG (H+L) (cat. no.715-606-150)	Jackson ImmunoResearch Inc. (West Grove, PA, USA)
Alexa Fluor® 647 AffiniPure F(ab′)$_2$ Fragment Donkey Anti-Goat IgG (H+L) (cat.no. 705-606-147)	Jackson ImmunoResearch Inc. (West Grove, PA, USA)
PE Goat Anti-Mouse Ig (Multiple Adsorption) (cat .no. 550599)	BD Pharmingen

Table S4. TaqMan primers used in the qPCR analysis.

Gene	Manufacturer	Primer Name
CAPN6	Thermo Fisher Scientific	Hs00560073_m1
GAPDH	Thermo Fisher Scientific	Hs02758991_g1
ITGA3	Thermo Fisher Scientific	Hs01076879_m1
ITGA6	Thermo Fisher Scientific	Hs01041011_m1
ITGA7	Thermo Fisher Scientific	Hs01056475_m1
ITGA10	Thermo Fisher Scientific	Hs00174623_m1
MGP	Thermo Fisher Scientific	Hs00969490_m1

References

1. Leloup, L.; Wells, A. Calpains as potential anti-cancer targets. *Expert Opin Ther Targets* **2011**, *15*, 309–323, doi:10.1517/14728222.2011.553611.
2. Fu, M.H.; Wang, C.Y.; Hsieh, Y.T.; Fang, K.M.; Tzeng, S.F. Functional Role of Matrix gla Protein in Glioma Cell Migration. *Mol Neurobiol* **2018**, *55*, 4624–4636, doi:10.1007/s12035-017-0677-1.
3. Mertsch, S.; Schurgers, L.J.; Weber, K.; Paulus, W.; Senner, V. Matrix gla protein (MGP): an overexpressed and migration-promoting mesenchymal component in glioblastoma. *BMC Cancer* **2009**, *9*, 302, doi:10.1186/1471-2407-9-302.
4. Chen, J.; Yuan, W.; Wu, L.; Tang, Q.; Xia, Q.; Ji, J.; Liu, Z.; Ma, Z.; Zhou, Z.; Cheng, Y., et al. PDGF-D promotes cell growth, aggressiveness, angiogenesis and EMT transformation of colorectal cancer by activation of Notch1/Twist1 pathway. *Oncotarget* **2017**, *8*, 9961–9973, doi:10.18632/oncotarget.14283.
5. Abdel-Malak, N.A.; Srikant, C.B.; Kristof, A.S.; Magder, S.A.; Di Battista, J.A.; Hussain, S.N. Angiopoietin-1 promotes endothelial cell proliferation and migration through AP-1-dependent autocrine production of interleukin-8. *Blood* **2008**, *111*, 4145–4154, doi:10.1182/blood-2007-08-110338.
6. Fiorio Pla, A.; Ong, H.L.; Cheng, K.T.; Brossa, A.; Bussolati, B.; Lockwich, T.; Paria, B.; Munaron, L.; Ambudkar, I.S. TRPV4 mediates tumor-derived endothelial cell migration via arachidonic acid-activated actin remodeling. *Oncogene* **2012**, *31*, 200–212, doi:10.1038/onc.2011.231.
7. Lee, W.H.; Choong, L.Y.; Jin, T.H.; Mon, N.N.; Chong, S.; Liew, C.S.; Putti, T.; Lu, S.Y.; Harteneck, C.; Lim, Y.P. TRPV4 plays a role in breast cancer cell migration via Ca(2+)-dependent activation of AKT and downregulation of E-cadherin cell cortex protein. *Oncogenesis* **2017**, *6*, e338, doi:10.1038/oncsis.2017.39.
8. Wen, L.; Wen, Y.C.; Ke, G.J.; Sun, S.Q.; Dong, K.; Wang, L.; Liao, R.F. TRPV4 regulates migration and tube formation of human retinal capillary endothelial cells. *BMC Ophthalmol* **2018**, *18*, 38, doi:10.1186/s12886-018-0697-2.
9. Kim, J.; Erikson, D.W.; Burghardt, R.C.; Spencer, T.E.; Wu, G.; Bayless, K.J.; Johnson, G.A.; Bazer, F.W. Secreted phosphoprotein 1 binds integrins to initiate multiple cell signaling pathways, including FRAP1/mTOR, to support attachment and force-generated migration of trophectoderm cells. *Matrix Biol* **2010**, *29*, 369–382, doi:10.1016/j.matbio.2010.04.001.
10. Matsuda, Y.; Miura, K.; Yamane, J.; Shima, H.; Fujibuchi, W.; Ishida, K.; Fujishima, F.; Ohnuma, S.; Sasaki, H.; Nagao, M., et al. SERPINI1 regulates epithelial-mesenchymal transition in an orthotopic implantation model of colorectal cancer. *Cancer Sci* **2016**, *107*, 619–628, doi:10.1111/cas.12909.
11. Liu, J.J.; Liu, J.Y.; Chen, J.; Wu, Y.X.; Yan, P.; Ji, C.D.; Wang, Y.X.; Xiang, D.F.; Zhang, X.; Zhang, P., et al. Scinderin promotes the invasion and metastasis of gastric cancer cells and predicts the outcome of patients. *Cancer Lett* **2016**, *376*, 110–117, doi:10.1016/j.canlet.2016.03.035.

© 2019 by the authors. Licensee MDPI, Basel, Switzerland. This article is an open access article distributed under the terms and conditions of the Creative Commons Attribution (CC BY) license (http://creativecommons.org/licenses/by/4.0/).
